# Unsaturated Glycerophospholipids Mediate Heme Crystallization: Biological Implications for Hemozoin Formation in the Kissing Bug *Rhodnius prolixus*

**DOI:** 10.1371/journal.pone.0088976

**Published:** 2014-02-26

**Authors:** Renata Stiebler, David Majerowicz, Jens Knudsen, Katia C. Gondim, David W. Wright, Timothy J. Egan, Marcus F. Oliveira

**Affiliations:** 1 Laboratório de Bioquímica de Resposta ao Estresse, Instituto de Bioquímica Médica Leopoldo de Meis, Universidade Federal do Rio de Janeiro, RJ, Brazil; 2 Laboratório de Inflamação e Metabolismo, Instituto Nacional de Ciência e Tecnologia de Biologia Estrutural e Bioimagem, Universidade Federal do Rio de Janeiro, Rio de Janeiro, RJ, Brazil; 3 Department of Chemistry, Vanderbilt University, Nashville, Tennessee, United States of America; 4 Laboratório de Bioquímica e Fisiologia de Insetos, Instituto de Bioquímica Médica Leopoldo de Meis, Universidade Federal do Rio de Janeiro, RJ, Brazil; 5 Department of Biochemistry and Molecular Biology, University of Southern Denmark, Odense, Denmark; 6 Departamento de Biotecnologia Farmacêutica, Faculdade de Farmácia,Universidade Federal do Rio de Janeiro, RJ, Brazil; 7 Instituto Nacional de Ciência e Tecnologia em Entomologia Molecular, Rio de Janeiro, RJ, Brazil; 8 Department of Chemistry, University of Cape Town, Private Bag, Rondebosch, South Africa; Swedish University of Agricultural Sciences, Sweden

## Abstract

Hemozoin (Hz) is a heme crystal produced by some blood-feeding organisms, as an efficient way to detoxify heme derived from hemoglobin digestion. In the triatomine insect *Rhodnius prolixus*, Hz is essentially produced by midgut extracellular phospholipid membranes known as perimicrovillar membranes (PMVM). Here, we investigated the role of commercial glycerophospholipids containing serine, choline and ethanolamine as headgroups and *R. prolixus* midgut lipids (RML) in heme crystallization. All commercial unsaturated forms of phospholipids, as well as RML, mediated fast and efficient β-hematin formation by means of two kinetically distinct mechanisms: an early and fast component, followed by a late and slow one. The fastest reactions observed were induced by unsaturated forms of phosphatidylethanolamine (uPE) and phosphatidylcholine (uPC), with half-lives of 0.04 and 0.7 minutes, respectively. β-hematin crystal morphologies were strikingly distinct among groups, with uPE producing homogeneous regular brick-shaped crystals. Interestingly, uPC-mediated reactions resulted in two morphologically distinct crystal populations: one less representative group of regular crystals, resembling those induced by uPE, and the other largely represented by crystals with numerous sharp edges and tapered ends. Heme crystallization reactions induced by RML were efficient, with a heme to β-hematin conversion rate higher than 70%, but clearly slower (t1/2 of 9.9–17.7 minutes) than those induced by uPC and uPE. Interestingly, crystals produced by RML were homogeneous in shape and quite similar to those mediated by uPE. Thus, β-hematin formation can be rapidly and efficiently induced by unsaturated glycerophospholipids, particularly uPE and uPC, and may play a role on biological heme crystallization in *R. prolixus* midgut.

## Introduction

Heme bound proteins are directly involved in a number of central cellular processes, including electron transfer, peroxide decomposition and gas transport [1]–[4]. However, in certain circumstances, heme can be released from hemeproteins and once in the “free” state, it can promote both signaling and deleterious effects [4]. In this regard, the amphipatic nature of this molecule is related to the presence of both charged propionate and hydrophobic vinyl side chains of the porphyrin ring, whereas the pro-oxidant effects are essentially due to the presence of the redox active iron atom [5]–[10].

Heme associates with different kinds of membranes and this interaction modulates the permeability and selectivity of these structures [6], [7], [11]. The binding of heme to phospholipid vesicles depends mostly on physico-chemical factors such as the temperature, polar head group surface charge and the lipid composition [12], [13]. Heme binding to vesicles containing positively charged phospholipid headgroups is favored, while negatively charged phospholipids not only reduce heme binding to phosphatidylcholine vesicles (PCV), but also increase heme dissociation from these structures [13]. Heme association to PCV also causes permeabilization of these structures even at low concentrations in reactions potentiated by cholesterol [7]. In red blood cells (RBCs), heme associates with phospholipid membranes [11] and causes rapid hemolysis in a mechanism independent of heme-induced lipid peroxidation [6], [14].

Blood-feeding organisms have to deal with the potential toxicity of heme. The mechanisms involved in heme sequestration comprise its binding to specific apo-hemoproteins [15], as well as its precipitation as unspecific aggregates [16]. Some organisms have a particular way to detoxify heme, by forming a dark brown crystal called hemozoin (Hz) [17]. This crystal was first described in the digestive vacuole of malaria parasites (*Plasmodium*) [18] and subsequently in triatomine insects [19], [20], in the helminth *Schistosoma* species [21] and others [22], [23], representing the major heme detoxification pathway in these organisms. Hz is chemically and structurally identical to a synthetic heme product called β-hematin [24], but the mechanisms involved in biological heme crystallization are still poorly understood. As far as is known, several components reported as catalysts of heme crystallization include proteins like the Heme Detoxification Protein (HDP) in *Plasmodium* [25] and α-glucosidase [26], [27] as well as several kinds of lipids [28]–[33] including those present in the midgut of triatomine insects [34]. In *Schistosoma* parasites, Hz crystals were found in close association to extracellular lipid droplets [35] and their isolated lipids were able to induce heme crystallization [36], [37].

The focus on neutral lipids and phospholipids has grown after several indications that Hz crystals were found in intimate contact with amphiphilic structures such as phospholipid membranes and lipid droplets (bodies or nanospheres) [26], [29], [37], [38], [39]. Also, biomimetic artificial hydrophilic-hydrophobic interfaces, by using mixtures of polar organic solvents with aqueous acid solutions, promptly induce β-hematin formation [28], [40]–[42]. Despite the general consensus over the contribution of lipids present in malaria parasite digestive vacuoles as important mediators of Hz formation, the underlying mechanism remains controversial [29], [39], [43]. Previous evidence [29], [43] has indicated the presence of neutral lipids within the *Plasmodium* food vacuoles, and their catalytic role in heme crystallization [28], [29], [31], [32]. In addition, the presence of neutral lipid droplets was reported within the parasite food vacuole, implicating these structures in Hz formation. However, the involvement of neutral lipid droplets on heme crystallization has recently been argued in reports using a combination of different electron microscopy and spectroscopic techniques [39], [44], [45]. In these reports, it was unequivocally shown not only that Hz crystals were not encapsulated in neutral lipid droplets in that compartment, but also that they were aligned in association with the surface of the digestive vacuole inner membrane, implying this structure as the nucleation site of Hz formation [44], [45]. Thus, the mode of heme interaction with lipid membranes seems a key aspect to mechanistically understand the process of biological heme crystallization. In this respect, *in vitro* experiments using myristoyl-glycerol have shown that β-hematin crystals grew parallel to the water surface, indicating that hydroxyl groups would play a central role by allowing oriented heme crystallization [46]. This concept was further strengthened by demonstrating that neutral lipid blend droplets were able to induce oriented β-hematin formation through a direct contact with lipid surface [47]. Based on this evidence, it seems logical to consider phospholipids (PLs) as natural components involved in Hz formation. In support of this, heme crystallization activities have been observed for phosphatidylcholine [28], [29], [37], phosphatidylethanolamine [28], [48], phosphatidylserine [49] and others [48], [49]. Very recently, Huy and colleagues have shown that heme crystallization mediated by a series of phospholipids is governed by gel-to-fluid transition, where only phospholipids in the liquid state under experimental conditions were able to induce β-hematin formation [38]. In addition, membranes were also found associated with β-hematin crystals produced *in vitro* by phosphatidylcholine vesicles [38]. Curiously, previous reports investigating the role of lipids in β-hematin formation, did not consider phospholipids as potential catalysts of β-hematin in their results, despite their unequivocal presence (see Figure 3D of ref. [43] and Figure 3C of ref [29]). Taken together, this evidence strongly supports the notion that amphipathic structures, particularly phospholipid membranes, could provide a suitable environment for β-hematin formation.

In the midgut of the triatomine insect *Rhodnius prolixus*, Hz formation is mediated by perimicrovillar membranes (PMVM) [34], phospholipid bilayers that cover the epithelial cells of the midgut [50], [51], with fewer integral proteins [15], [16] and with phosphaditylethanolamine (PE) being the most abundant phospholipid synthesized by the midgut [52]. Hz crystals have also been observed in close association with PMVM [35], [20]. Previous data from our group have shown that lipids isolated from PMVM are efficient catalysts of Hz formation [30]. Thus, considering that phospholipids represent one of the dominant class of lipids found in *R. prolixus* midgut luminal content [53], in the present work, we investigated the contribution of phospholipids in chemical and biological heme crystallization.

## Materials and Methods

### 2.1.Chemicals and reagents

Hemin chloride was purchased from Frontier Scientific (Logan, USA). Commercial phospholipids (1,2-dilinoleoyl-sn-glycero-3-phosphoethanolamine) uPE 36:4, (1,2-dilinoleoyl-sn-glycero-3-phosphocholine) uPC 36:4 and (1,2-dioleoyl-sn-glycero-3[phospho-L-serine]) uPS 36:2 were obtained from Avanti Polar Lipids Inc. (Alabaster, USA). Pyridine, sodium bicarbonate, sodium carbonate, sodium hydroxide, glacial acetic acid, SDS, sodium citrate, HEPES (4-(2-hydroxyethyl)-1-piperazineethanesulfonic acid) and other reagents were obtained from Merck (Darmstadt, Germany). Chloroquine and quinine were from Sigma-Aldrich (St. Louis, MO, USA). All other reagents were of analytical grade. The water used in the study was of ultrapure grade.

### 2.2 Ethics statement

The animal care and experimental protocol was conducted following the guidelines of the institutional care and use committee (Ethics Committee for Animal Use from the Federal University of Rio de Janeiro, CEUA-UFRJ) and the NIH Guide for the Care and Use of Laboratory Animals (ISBN 0-309-05377-3). The protocol was approved by CEUA-UFRJ under registry #IBQM050. Technicians dedicated to the animal facility at the Institute of Medical Biochemistry (IBqM-UFRJ) carried out all aspects related to rabbit husbandry under strict guidelines to insure careful and consistent handling of the animals.

### 2.3. Animals

Adult *R. prolixus* females were reared at 28°C and 80% relative humidity, fed on rabbit blood or plasma using artificial feeders [54] and maintained in a colony at Federal University of Rio de Janeiro. Groups of insects (2^nd^ feeding cycle as adult) were fed with either rabbit blood or plasma and four days later, were dissected to obtain the midgut content. This was accomplished by incubating the midguts in plastic tubes containing 4 mL of cold phosphate buffered saline and a cocktail of protease inhibitors (Sigma, MO, USA) and gently shaken every 5 min. Then, the tubes were left undisturbed for 5 min to sediment the tissue debris and a supernatant aliquot was centrifuged at 20,000×*g* for 10 min at 4°C. The pellet fraction was collected, re-suspended in 200 µL of PBS and kept frozen until further analyses.

### 2.4. Lipids

Total lipids were extracted from *R. prolixus* midgut contents from plasma or blood-fed insects using a chloroform:methanol:aqueous solution (2∶1∶0.8 v/v) mixture, according to a previous method [55], to produce both *Rhondius* midgut lipid (RML) samples (plasma and blood).

### 2.5. Heme crystallization assay

To assess the role of phospholipids in heme crystallization, commercial phospholipids and biological lipids (previously extracted from *R. prolixus* adult female midgut contents) were firstly diluted in acetone:methanol 1∶9 (v/v) and incubated in 50 mM sodium citrate buffer pH 4.8 to reach final concentrations of 100 µM or 10 µg/mL, respectively over 24 h. Heme, previously prepared in 0.1 M NaOH from a 10 mM stock solution, was added to a final concentration of 100 µM. Reactions were carried out in polypropylene tubes at 28°C in a final volume of 200 µL, and the tubes were gently shaken throughout the incubation period (up to 24h). Reactions were stopped by adding 40 µL of an aqueous pyridine solution (30% pyridine, 10% HEPES buffer 2.0 M, pH 7.5, 40% acetone and 20% water v/v) to reach a final pyridine concentration of 5% (v/v). The sample was immediately centrifuged for 10 minutes at 4000 rpm. In the kinetics assay, the percentage of unconverted heme was determined colorimetrically at 405 nm on the supernatant of quenched and centrifuged samples. This is a modification of an assay previously published [31], [56], [57]. We also investigated the inhibitory effect of quinolines on heme crystallization reactions induced by *R. prolixus* midgut lipids. To accomplish this, the reactions were carried out in polypropylene tubes in the presence of 0.5 M sodium acetate buffer, pH 4.8, 100 µM hemin, previously prepared in 0.1 M NaOH as 10 mM stock solutions, with a final volume of 1.0 mL. A sample corresponding to 40 µg of total lipids previously extracted from *R. prolixus* adult female midgut contents was incubated for 24 h at 28°C in the presence or absence of 100 µM chloroquine (CLQ) or quinine (QUI). After incubation, the reaction mixture was centrifuged at 17,500×*g* for 10 min at room temperature. The pellet was washed three times with “extraction buffer” (0.1 M sodium bicarbonate and SDS 2.5%, pH 9.1), and twice with milliQ water. The final pellet was solubilized in 0.1 M NaOH and the amount of heme determined colorimetrically at 400 nm [58].

### 2.6. Characterization of β-hematin products

Fourier-Transformed Infrared (FTIR) spectroscopy was used to confirm the identity of β-hematin crystals produced by different lipids. Dried samples from β-hematin reactions incubated for 24 h were homogenized as Nujol mulls and the FTIR spectra were recorded between 2000 cm^−1^ and 1000 cm^−1^. Transmission Electron Microscopy (TEM) of β-hematin crystals produced by different lipids were carried out as follows: heme crystallization was induced by different lipids as described above for 24 h and after that 10 µL of the reaction mixture was applied onto a copper grid (400 mesh), coated with Formvar (TED Pella, Inc., Redding, USA). After 15 minutes, the excess sample was removed with filter paper. A 5% uranyl acetate solution was previously prepared by diluting 50 mg/mL uranyl acetate in water and left stirring for 12 h in the dark. The pH was adjusted with glacial acetic acid to 4.2–4.5 and then the sample was filtered with a syringe filter (0.22 µm). Thereafter, 5 µL of 5% uranyl acetate solution was applied to the sample and the excess solution was absorbed with filter paper. All samples were observed in a Philips CM20 transmission electron microscope.

### 2.7. Electron spray ionization mass spectrometry analyses of R. prolixus midgut phospholipids

Insects were dissected at the seventh day after blood meal and the posterior midguts were obtained. Lipids were extracted [55], solvent was vacuum dried and lipids were re-suspended in 500 µL methanol-chloroform (2∶1 by volume). Samples were centrifuged at 4,500×*g* and 4°C for 10 min and then diluted 10 times in the same solution. Aliquots (10 µL) were added to 12.9 µL of 1.33 mM ammonium acetate in propyl alcohol and run on a LTQ Orbitrap XL Mass Spectrometer (Thermo Fisher Scientific, Waltham, USA) equipped with a robotic nanoflow ion source TriVersaNanoMate (Advion Biosciences, Ithaca, USA) in positive and negative modes as described elsewhere [59]. Results were analyzed using Qual Browser software (Thermo Fisher Scientific, Waltham, USA) and Limsa add-on [60].

### 2.8. Data analyses

Data in graphs were presented as mean ± SD values for each condition. D'Agostino and Pearson normality tests were done for all values to assess their Gaussian distribution. When Gaussian distribution was achieved, outlier values were excluded by performing the Grubbs' test using the online tool available at http://graphpad.com/quickcalcs/Grubbs1.cfm. Comparisons between groups were done by Kruskal-Wallis and *a posteriori* Dunn's multiple comprarison test (for values without Gaussian distribution). Differences of *p*<0.05 were considered to be significant. Kinetics of β-hematin reactions were analysed by using the Avrami equation (Eq. 1) [61], 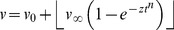
(1) where *v* is the amount of β-hematin formed (in nmols), *v*_0_ is the amount of β-hematin present at the beginning of the reaction, ν_∞_ is the amount of β-hematin formed at completion of the reaction, *z* is the rate constant and *n* is the Avrami constant. For a process in which growth occurs at an interface between the two interconverting phases, as is likely to be the case for β-hematin formation in this model reaction, n takes an integer value ranging between 1 and 4. All graphs and statistics were carried out by using the Prism software version 5.0 for Windows (GraphPad Software, USA).

## Results and Discussion

**Figure 1:**
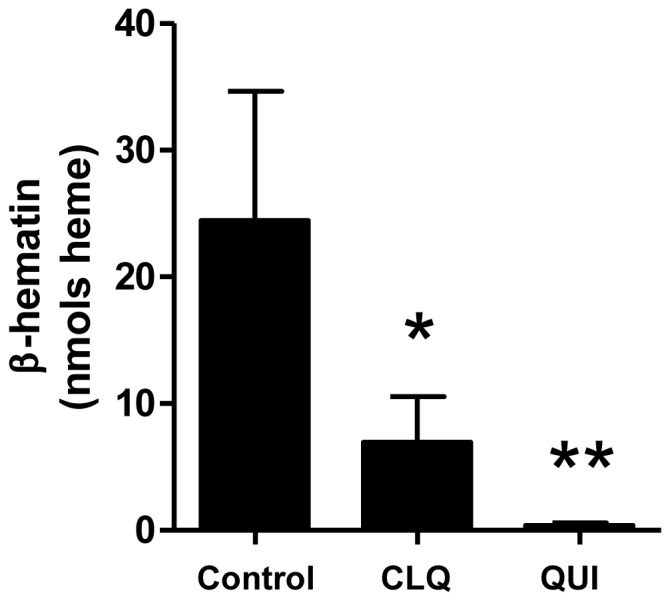
Lipids derived from *R. prolixus* midgut content promote heme crystallization in quinoline-sensitive reactions. Lipids were isolated from *R. prolixus* midgut content as described in the methods section and 40 µg of lipids were incubated for 24 h at 28°C in the presence of 100 µM heme alone (control), with 100 µM chloroquine (CLQ) or with 100 µM quinine (QUI). Data are expressed as mean ± SD of at least seven different experiments. Comparisons between groups were done by Kruskal-Wallis and *a posteriori* Dunn's tests. **p<0.0001 (control *vs.* QUI) and *p<0.05 control *vs.* CLQ).

Previous work has shown that in triatomine insects Hz crystals are found in close association with a particular kind of phospholipid bilayer, namely perimicrovillar membranes (PMVM) [20], [26], [30], [34], [35]. The activity responsible for Hz formation in *R. prolixus* midgut was found to be associated with a particular fraction of the midgut content, which was not derived from the actinomycete endosymbiont *Rhodococcus rhodnii* [34]. Also, chloroquine was able to efficiently inhibit heme crystallization *in vitro* and *in vivo* leading to redox imbalance and molecular damage to the insect hemolymph [34]. Later, Silva and colleagues demonstrated that Hz formation in *R. prolixus* midgut was directly correlated with α-glucosidase activity, a marker enzyme of PMVM [26], [62]. More recently, our group has shown that total lipids isolated from midgut content were able to promptly stimulate formation of β-hematin crystals *in vitro*, regardless of insect diet [30]. Thus, our first approach was to determine whether heme crystallization promoted by lipids isolated from *R. prolixus* midgut would be inhibited by quinoline antimalarials. Figure 1 shows that total lipids isolated from *R. prolixus* midgut were able to promote heme crystallization in reactions inhibited by both chloroquine (71.5%) and quinine (91%). This result is in agreement with a previous report showing that quinolines efficiently inhibited phospholipid-mediated β-hematin formation [48]. In fact, it was recently shown that PLs represent not only one of the dominant class of lipids found in *R. prolixus* midgut luminal content [53] but also the vast majority of lipids synthesized by midgut epithelium (80% of all lipids) [52]. Based on these data, we designed a set of experiments utilizing both commercial phospholipids (Table 1) and biological lipids obtained from *R. prolixus* midgut to assess their potential role in heme crystallization.

**Table 1:** Structural and physico-chemical features of the glycerophospholipids investigated in the present work.

Chemical name	Abbreviations	T_m_ a	Molecular formula	Acyl groups
1,2-dilinoleoyl-sn-glycero-3-phosphocholine	uPC	−53°C	C_44_H_80_NO_8_P	36:4 (18:2/18:2)
1,2-dioleoyl-sn-glycero-3[phospho-L-serine]	uPS	−11°C	C_42_H_77_NO_10_PNa	36:2 (18:1/18:1)
1,2-dilinoleoyl-sn-glycero-3-phosphoethanolamine	uPE	−40°C	C_41_H_74_NO_8_P	36:4 (18:2/18:2)
1,2-dipalmitoyl-sn-glycero-3-phosphocholine	sPC	42°C	C_40_H_80_NO_8_P	32:0 (16:0/16:0)
1,2-dipalmitoyl-sn-glycero-3-phosphoethanolamine	sPE	63°C	C_37_H_74_NO_8_P	32:0 (16:0/16:0)

a T_m_ values were obtained from the literature [67].

Table 1 shows that all five commercial phospholipids investigated in the present study are glycerophopholipids with choline (PC), serine (PS) and ethanolamine (PE) as headgroups, and differing little in terms of the acyl side chain size and unsaturation. Two out of the five phospholipids tested, saturated PC (sPC) and saturated PE (sPE), contain no double bonds in their acyl chains, which would be expected to directly affect their heme crystallization activity, as their phase transition temperatures (T*_m_*) are above of the experimental temperature conditions in this present work. This is an important feature to consider, since it was recently demonstrated that the main physico-chemical factor responsible for heme crystallization activity mediated by phospholipids is the T*_m_*. Since this parameter is affected not only by the size but also by the unsaturation of the acyl chains, as well as the polar headgroup [38], it is expected that small structural changes would be reflected in heme crystallization. Thus, we expected that the unsaturated phospholipids tested in the present work (uPC, uPS, and uPE), would be in liquid state under our experimental conditions, since their phase transition temperatures (T*_m_*) are far below the reaction temperatures used in our experiments (Table 1). On the other hand, the saturated phospholipids would be in gel state as their T*_m_* values are above of the experimental temperature, thus affecting their heme crystallization activity (Table 1 and [38]). As expected, the saturated phospholipids (sPC and sPE) were unable to promote β-hematin formation in reactions conducted at 28°C in citrate buffer pH 4.8 (data not shown), which could be a direct consequence of their high T*_m_* values, arising from the gel state of both phospholipids. However, we must concede that besides the presence and number of unsaturations, physico-chemical conditions such as temperature, pH and salinity can directly influence the T*_m_* value. It must therefore also be considered that all T*_m_* values reported in the literature were defined at pH and ionic strength quite distinct from that of our experimental conditions, which could also affect this parameter and then their heme crystallization activity.

**Figure 2:**
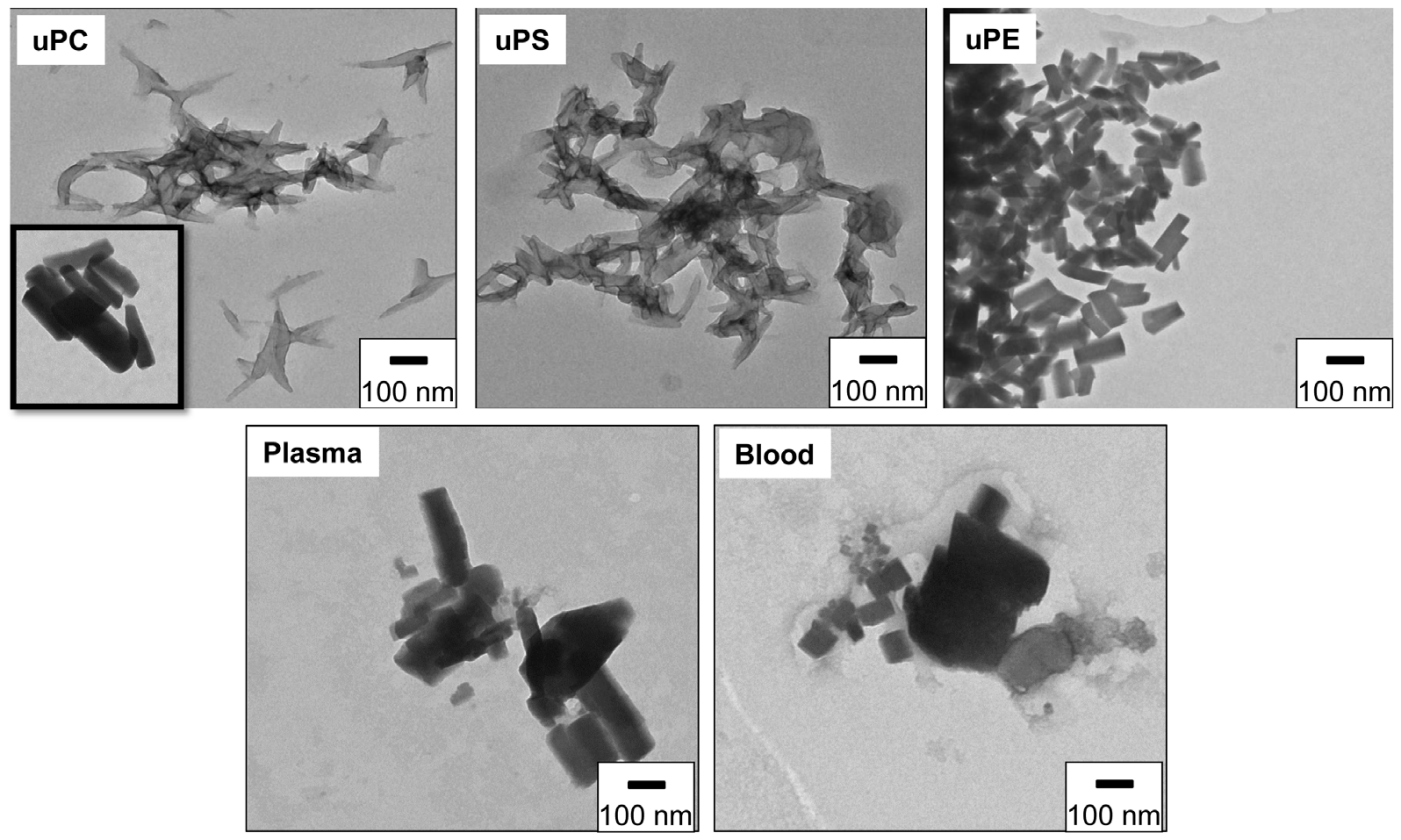
Unsaturated phosphatidylethanolamine produced homogeneous crystals morphologically similar to those induced by *R. prolixus* midgut lipids. Transmission electron microscopy of crystals induced by 100 µM uPC (A), uPS (B) or uPE (C) or 10 µg/mL total lipids isolated from *R. prolixus* midgut content previously fed with plasma (D) or blood (E). The inset shown in uPC represent a very small population of regularly shaped crystals produced by uPC. Scale bars represent 100 nm for all images, including the inset.

**Figure 3:**
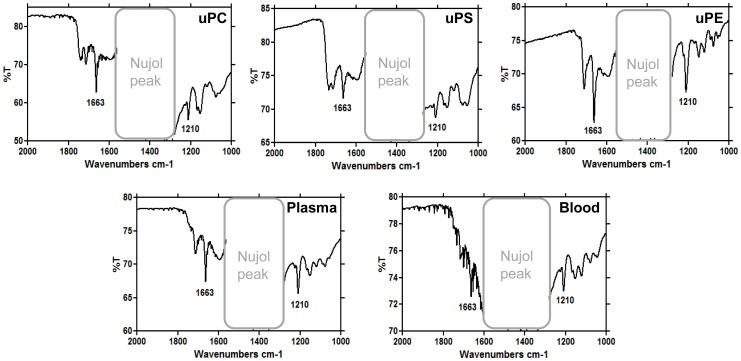
Fourier transformed infrared spectroscopy identifies the crystals produced by different lipids as β-hematin. The crystals were produced by 100 µM uPC (A), uPS (B), uPE (C) or 10 µg/mL total lipids isolated from PMVM of *R. prolixus* previously fed with plasma (D) or blood (E). The characteristic iron-carboxylate peaks of β-hematin at 1210 and 1663 cm^−1^ are shown.

The external morphologies of crystals produced by commercial phospholipids and biological lipids were investigated by transmission electron microscopy (TEM). Figure 2 shows images of crystals produced by uPC, uPS and uPE, demonstrating the extreme diversity of external morphologies induced by these phospholipids. In general, the purified crystalline reaction products from uPC and uPS were more irregular in shape and with numerous sharp edges and tapered ends, strikingly differing from those obtained from uPE or *R. prolixus* midgut lipids. On the other hand, the crystals produced by uPE were homogeneously very regular in shape, strongly resembling those produced by the lipids from *R. prolixus* midgut. Interestingly, heme crystallization mediated by uPC was heterogeneous since we could detect a very small population (less than 5%) of regular shaped crystals in the uPC samples (Figure 2, *inset* of uPC), which were morphologically undistinguishable to those produced by uPE and *R. prolixus* midgut lipids. Conceivably, these morphological differences would be explained by the ways the which heme molecules interact with the polar headgroups of uPC (CH_3_) and uPE (OH). Interestingly, previous experiments using self-assembled functionalized alkanethiol monolayers exposing OH and CH_3_ groups [46] demonstrated that surfaces exposing OH groups promoted heme crystallization essentially through the {100} crystal face, whereas in CH_3_ groups crystal growth was observed in both {100} and {010} faces, but preferentially to {010} face. β-hematin crystals produced by lipids from plasma-fed or blood-fed insects were brick shaped with blunt ends, being quite similar among each other and to those induced by uPE (Figure 2, uPE) as well as the small population of regular crystals from uPC (Figure 2, *inset* of uPC). It is interesting to observe the strong morphological similarity of crystals from uPE and *R. prolixus* midgut lipids, which can be explained by the fact that PE represents the major phospholipid synthesized by *R. prolixus* midgut epithelia [52]. This suggests that uPE, and to a lesser extent uPC, would play an important role in determining Hz crystal morphology in *R. prolixus* midgut, when compared to other midgut phospholipids. Noteworthy, the insect diet composition seemed not to define the shapes or sizes of β-hematin crystals, since midgut lipids obtained from plasma and blood fed insects produced morphologically similar heme crystals (Figure 2). Despite the fact that crystals produced by different catalysts in Figure 2 exhibited quite distinct morphologies, all of them have the characteristic FTIR Hz-specific transmittance peaks at 1210 cm^−1^ and 1663 cm^−1^, confirming the identity of these products as genuine β-hematin crystals (Figure 3).

**Figure 4:**
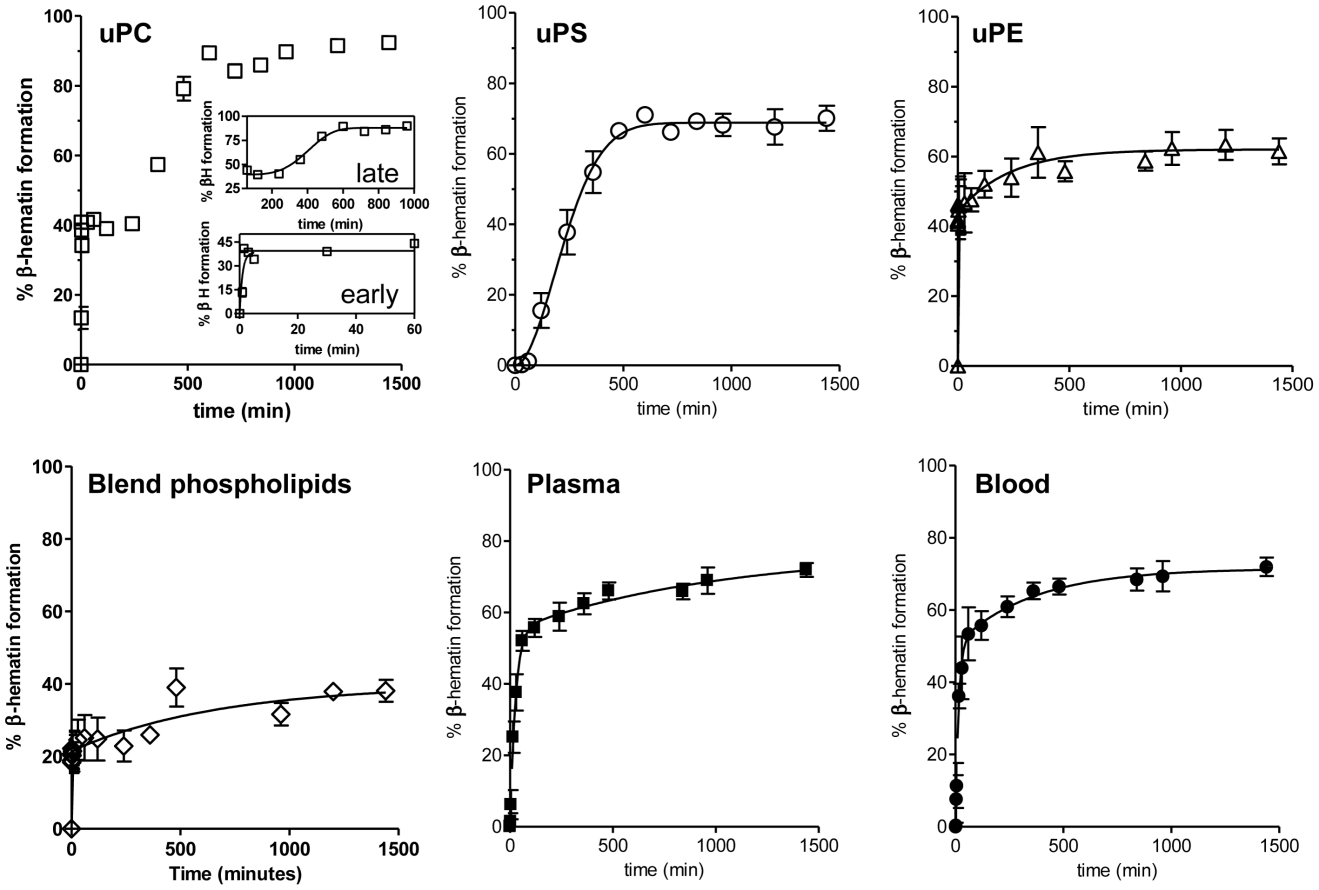
Kinetics of heme crystallization promoted by different commercial and biological lipids. Heme crystallization reactions were induced *in vitro* mediated by uPC, uPS or uPE (100 µM), a blended phospholipid mixture of commercial uPS (14%), uPC (32%) and uPE (51%) or 10 µg/mL of total lipids isolated from PMVM of *R. prolixus* previously fed with plasma or blood. Data are expressed as mean ± SD, of at least three different experiments and fitted using the Avrami equation as described in the methods section. To perform the Avrami analysis, the uPC-induced kinetics were independently analyzed at early and late times, which are shown as insets.

The kinetic profiles of β-hematin formation mediated by commercial unsaturated phospholipids and *R. prolixus* midgut lipids were determined along 24 h of reaction (Figure 4). For this set of experiments we also included a blend of uPE (51%), uPC (32%) and uPS (14%), in an attempt to reach similar ratios of these phospholipids to those synthesized by *R. prolixus* midgut [52]. A brief visual inspection of the fitted curve shapes for all three commercial phospholipids, indicates that all reactions mediated fast and efficient β-hematin formation with quite distinct kinetic patterns among the groups (Figure 4). Reactions induced by uPC clearly exhibited two kinetically distinct components, both contributing substantially to crystal formation: a very fast early process, converting about 40% of heme into β-hematin and a slower late one, which converts further 48%. It is tempting to suggest that the kinetic behavior of the reaction induced by uPC may be related to the two distinct crystal morphologies exhibited by this phospholipid (Figure 2, uPC). Curiously, the brick-shaped crystals produced by uPC are quite similar to those of uPE, while the needle-like ones resemble the uPS crystals. However, this suggestion does not correlate with the very small fraction of the brick-like crystals produced in this process. Another possibility to consider is that these two kinetically distinct components of uPC were related to the way by which heme interacts with the exposed CH_3_ groups at the surface of uPC vesicles [46]. Interestingly, β-hematin formation induced by the phospholipid blend was revealed to be as fast as the uPE-mediated heme crystallization, suggesting that beyond representing the dominant phospholipid synthesized by *R. prolixus* midgut epithelia [52] uPE is able to produce crystals very similar in shape to those found biologically (Figure 2), mediating fast and efficient β-hematin formation (about 60% of heme conversion) (Figure 4). Regardless of the catalyst, we can observe that all reactions were essentially completed after 8 h (∼500 minutes) and all exhibited two kinetically distinct components: one very fast, making a major contribution to β-hematin conversion (54% to 100%), and the other a slow one. With the exception of uPC, this slow component does not greatly contribute to the overall heme conversion to β-hematin and its mechanism is uncertain. Therefore, in Table 2 we show five distinct calculated kinetic parameters of reactions induced by commercial and biological lipids, based on their pattern shown in Figure 4. This was accomplished by fitting all seven sets of kinetics data to the Avrami equation (see “*data analyses*” in methods section), which mathematically describes the processes of solid transition from one phase to another at constant temperature [61]. Since all data could be fitted to the Avrami equation, this indicates that β-hematin formation mediated by the different lipids involves both nucleation and growth. The Avrami constant, *n*, can only take on integer values between 1 and 4 and this gives an insight into the geometry of crystal growth and the nature of the nucleation process, which could be instantaneous (all nuclei are preformed at the beginning of the process), or sporadic (nuclei form throughout the process). When n = 1, which is the case for all catalysts, with exception of the late uPC component and uPS, the fits are mathematically indistinguishable from first-order processes, with exponential curves, which can be interpreted to mean that they occur through one dimensional growth at preformed nuclei. Firstly, heme conversion to β-hematin varied from 39.8% with Blend to 96.5% with the overall uPC-mediated reactions, while in lipids from *R. prolixus* midgut this conversion was about 74%. We speculate that the different sizes of vesicles produced by the phospholipids may explain the discrepancies of heme conversion to β-hematin. This possibility is supported by literature [31], [39] which has proposed that the β-hematin crystal nucleates at the surface of neutral lipid particles [31] or DV membranes [39], [44], [45] and grows along the lipid-water interface until the curvature of the lipid particle limits this process. Conceivably, uPC would produce the largest vesicles, and the Blend the smallest ones. Regarding the rate constants (*z*), the data obtained for uPS and the late component of uPC (Table 2) cannot be compared to other phospholipids because of the different value of the Avrami constant *n*, and hence different units. Nevertheless, it is remarkable to note the differences in reaction half-lives mediated by uPE (0.04 min.) which are orders of magnitude lower than those of uPC (0.7 min., for early, and 402 min., for the late component), uPS (225 min.), and those induced by lipids from plasma or blood fed *R. prolixus* biological lipids (17.7 and 9.9 min., respectively). Interestingly, the reaction half-life mediated by uPE was undistinguishable from those of reactions promoted by phospholipid blend (0.04 *vs*. 0.035 min., respectively, *p* = 0.88). Previous reports have demonstrated that phospholipids are efficient catalysts of heme crystallization [28], [48], with variable results. For instance, the study conducted by Dorn and colleagues demonstrated that PE, PS, PC, phophatidylinositol (PI) and sphingomyelin were all able to produce β-hematin, with PC being more efficient than PE in overnight reactions [48]. However, the opposite was shown in a study of Egan and colleagues, where the heme conversion to β-hematin mediated by PE was higher than PC, after only five minutes of reaction [28]. Since the exact fatty acid composition of phospholipids in both reports are unknown, a direct comparison between these data and our results cannot be made. Potentially, the kinetic behavior of uPE and uPC in mediating β-hematin formation may explain the relative quickness and extent by which heme crystallization reactions proceed in plasma and blood derived *R. prolixus* midgut lipids, considering that this preparation would contain uPE, uPC and uPS in different proportions [52]. Also, these data highlight the effective contribution of each phospholipid in the reaction process, in which the fast component of uPC and uPE would play a central catalytic role at early time points of β-hematin formation, whereas the slower uPC component would play a prominent role at later reaction times, increasing the conversion extent. An alternative explanation for the differences observed in the rate constant and half-life of the three phospholipids tested could be their effect in reducing the activation energy required for heme transition from the π-π dimer to reciprocal iron-carboxylate dimers of β-hematin. Conceivably, the extent of the reduction of the activation energy barrier for this transition would be provided by the chemical environment of each phospholipid headgroup, thus affecting the kinetics of reaction.

**Table 2:** Fitted kinetic parameters for β-hematin formation mediated by commercial phospholipids and biological lipids from *R. prolixus* midgut.

Lipid	% overall conversion	% contribution to overall reaction	n	Rate constant (z)	Half-life (minutes)
uPC (early)	38±3	44±3	1	1.0±0.3 min^−1^	0.7±0.2
uPC (late)	88±2	66±3	4	2.7±0.3×10^−11^ min^−4^	402±12
uPS	68.8±0.8*^a^*	100	2	0.00002±0.00001 min^−2^	225±10
uPE	62±1*^a,c^*	71± 2	1	17.1±5.1 min^−1^	0.04±0.01^*^
Blend	39.8±3.8*^a,d,e^*	54±5	1	19.7±16.6 min^−1^	0.035±0.030^*^
RML plasma	77.0±6.5*^b,f,g^*	72±5	1	0.039±0.003 min^−1^	17.7±1.4
RML blood	71.5±1.6*^b,f,g^*	71± 3	1	0.070±0.008 min^−1^	9.9±1.1

Values were expressed as mean ± SEM of five distinct kinetic parameters obtained from the data shown in Figure 4. Statistical analyses between groups were performed by using the Mann-Whitney (superscript letters) or Student's t tests (superscript symbols). *^a^p*<0.05 relative to uPC; *^b^p*<0.01 relative to uPC; *^c^p*<0.05 relative to uPS; *^d^p*<0.005 relative to uPS; *^e^p*<0.005 relative to uPE; *^f^p*<0.001 relative to uPE; *^g^p*<0.0005 relative to RML plasma. Comparisons of reactions half-lives showed that all groups were statistically distinct among each other (*p*<0.03), with exception of the uPE and Blend comparison (*p* = 0.88).

Figure 4 also shows that the kinetic profile of heme crystallization reactions induced by lipids from the midgut of plasma and blood fed insects were very fast (17.7 and 9.9 min., respectively) when compared to uPS and late uPC (225 and 402 min., respectively), but clearly slower than early uPC, uPE and blend-mediated reactions (0.7, 0.04 and 0.035 min., respectively). This indicates that, regardless the insect diet, lipids from midgut content are able to rapidly induce β-hematin formation (Figures 2 and 3), extensively producing crystals (77% conversion rate, Table 2) with very regular shape (Figure 2). We think that slower reactions catalyzed by *R. prolixus* midgut lipids when compared to uPC and uPE may be explained by the presence of other components in those samples, such as contaminant proteins (albumin, for example) that would loosely bind heme and compete with this molecule, avoiding their interaction to form β-hematin, or even to cholesterol that also modulates heme partitioning into biological membranes [7]. It must also be taken into account that the fatty acid structures present in *R. prolixus* midgut phospholipids might not be the same as those present in the commercial phospholipids tested in the present work. Thus, in order to investigate the phospholipid composition of *R. prolixus* midgut, a mass spectrometry analysis was carried out on whole lipids isolated from that compartment. Table S1 shows that the dominant acyl chains of phospholipids present in *R. prolixus* midgut were uPE 36:1 (38.0%), uPS 40:1 (43.6%), and uPC 36:2 (61.3%). Essentially, we can observe that all phospholipids found in *R. prolixus* midgut exhibit acyl chains from 34 to 40 with few unsaturations (1 to 3), with exception of PS 38:0, which would reflect a negative T*_m_* for all phospholipids. Thus, since the acyl chains differ little among commercial phospholipids and those from biological origin, this seems not to explain the differences observed in both crystal morphology and kinetics of β-hematin formation, suggesting that these reactions are essentially governed by the structure of phospholipid headgroups. Strengthening this concept, the differences observed in the kinetic parameters of uPC and uPE mediated heme crystallization (Figure 4 and Table 2) as well as on the crystals morphologies (Figure 3), can only be ascribed to choline and ethanolamine, respectively, since both have identical acyl chains (Table 1). Remarkably, both morphology and kinetic profiles of β-hematin crystal formation are quite similar, when the uPE and biologically-mediated reactions are compared (Figures 2 and 4). This assumption is strengthened when one considers that heme binding to phospholipid membranes varied little with fatty acid chain length or saturation, as long as the measurements were made at temperatures well above those for the liquid-gel phase transition [13]. Therefore, it seems that the slight changes in fatty acid structure of the phospholipids tested here may not explain the observed results, since their T*_m_* were all far below the temperatures of reactions conducted in our experiments [13]. Although true T*_m_* values of *R. prolixus* phospholipids identified in Table S1 are currently unknown, we can only speculate about the implications of their structures on biological heme crystallization. Certainly, this is a limitation of our study, since we cannot directly extrapolate the results of heme crystallization with commercial phospholipids to biological lipids. Rather, we can compare both samples and discuss their roles in kinetics, catalysis and crystal growth.

Since the phospholipid headgroups seems to be involved in heme crystallization, it is important to consider their physico-chemical features, especially their p*K*_a_, as potential explanations for differences in β-hematin formation kinetics and crystal morphologies. In this regard, we assumed that PS has an overall negative charge as the p*K*_a_ of its phosphate is 2.6 and the carboxylate group is 5.5, which is quite near the pH of β-hematin reactions conducted chemically (pH = 4.8) or biologically [63]. Previous observations have shown that the presence of negative charges decreased the binding of heme to liposomes, supporting the notion that electrostatic repulsion limits heme transfer to liposomes and increased their dissociation rate from these structures [13], [64]. Maybe, the presence of the negative charges of PS, as a result of phosphate and carboxylic acid dissociation at physiological pH (4.8), would promote charge repulsion of these groups with the dissociated propionate groups of heme, explaining the slower reactions catalyzed by this phospholipid in our experiments. The single ionizable group of choline in PC is the phosphate with an apparent p*K*_a_ of 1.0 [63], whereas in PE the ethanolamine head group has two ionizable groups, the phosphate and the amino groups, with quite distinct p*K*_a_s (1.7 and 9.6, respectively) [63]. This strongly indicates that overall charge of phospholipid headgroup of PE cannot explain the differences in both crystal morphology and kinetics observed in our work and other types of interactions may take place between heme and PE which are not currently known. On the other hand, the protonated NH_3_^+^ group of PE would be capable of hydrogen bonding to the carboxylate group of heme, whereas the N(CH_3_)_3_^+^ group of PC cannot. This may be a crucial factor in the activity of PE relative to PC. Further research is required to better understand the nature of heme-phospholipid interaction in the context of β-hematin formation.

Much evidence in the literature has shown that lipids play an important role as biological catalysts of heme crystallization [17], [26], [28]–[33], [35]–[48], [65], [66], from *Plasmodium*-derived digestive vacuole lipids [32], [33], [39], [43]–[45], [47] to *Schistosoma*-derived lipids [35]–[37] and *R. prolixus*-derived lipids (reference [30], and Figures 1–4). Moreover, rapid and efficient crystallization of heme *in vitro* can be achieved through a hydrophilic-hydrophobic interface formed by artificial neutral lipid constituents of the digestive vacuole [28]. Interestingly, we have demonstrated that only four days after blood feeding at least 97% of all iron species found in *R. prolixus* midgut are present as Hz [30]. This strongly indicates that whatever the mechanism involved in Hz formation *in vivo*, it is a very efficient process. Thus, based on the data presented here, we suggest that a combination of uPE and uPC may play a role in Hz formation in this organism. Importantly, given the fast kinetics of heme crystallization mediated by uPE and lipids from *R. prolixus* midgut, and the striking morphological similarity of β-hematin crystals produced by these samples, it is hard to conceive the need of a protein, such as α-glucosidase from *R. prolixus* or HDP from *Plasmodium* to induce biological Hz crystallization. We postulate that in *R. prolixus* midgut after hemoglobin is digested by proteases, heme is released and diffuses through the lumen until reaches the PMVM, where it interacts with the headgroups of the constituent phospholipids. This interaction would be facilitated by heme transfer from aqueous medium, possibly as π-π dimers, to the PMVM surface, where heme accumulates thereby reducing the activation energy required for heme transition from this species to reciprocal heme dimers of β-hematin.

In conclusion, the present study demonstrates that unsaturated glycerophospholipids, particularly uPE and uPC, are true catalysts of β-hematin formation, mediating fast and efficient heme crystallization. Given the similarities observed in reaction kinetics and the crystal morphologies, it is plausible that uPE and uPC may be involved in heme crystallization in *R. prolixus* midgut. These data, together with literature implicating the involvement of phospholipids in β-hematin formation [30], [38], represent a significant step towards understanding the mechanisms involved in heme crystallization and may open new perspectives for rational intervention in this process.

## Supporting Information

Table S1 **Glycerophospholipid composition found in blood fed** ***R. prolixus*** **midgut determined by mass spectrometry.**
(DOCX)
