# Supplementary material for: Unsaturated Glycerophospholipids Mediate Heme Crystallization: Biological Implications for Hemozoin Formation in the Kissing Bug Rhodnius prolixus
Source: PLoS One. 2014 Feb 26;9(2):e88976. doi: 10.1371/journal.pone.0088976 (PMC3935856; doi:10.1371/journal.pone.0088976)
Supplement: Table S1 — Glycerophospholipid composition found in blood fed R. prolixus midgut determined by mass spectrometry. (DOCX) [file pone.0088976.s001.docx]

**Table S1: Glycerophospholipid composition found in blood fed *R. prolixus* midgut determined by mass spectrometry.**

| **Glycerophospholipid specie** | **Relative amount (% ± SEM)** |
| --- | --- |
| **Phosphatidylethanolamine (PE)** |  |
| 34:1 | 28.7 ± 2.45 |
| 36:1 | 38.0 ± 0.58 |
| 36:2 | 20.6 ± 5.15 |
| **Phosphatidylserine (PS)** |  |
| 36:1 | 6.26 ± 0.97 |
| 36:2 | 13.3 ± 3.79 |
| 38:0 | 14.6 ± 5.76 |
| 38:1 | 7.14 ± 0.87 |
| 40:1 | 43.6 ± 8.96 |
| **Phosphatidylcoline (PC)** |  |
| 34:1 | 13.9 ± 1.00 |
| 34:2 | 5.91 ± 1.15 |
| 36:2 | 61.3 ± 0.59 |
| 36:3 | 5.24 ± 1.30 |

Values represent the relative amount of each glycerophospholipid as mean ± SEM of four distinct experiments. The results represent only the phospholipids class from all lipids analyzed in *R. prolixus* midgut and comprise only those species that represent at least 5% from total phospholipids. Each glycerophospholipid species are denoted as "total number of carbon in all fatty acids chains":"total number of double bonds in all fatty acids chains".
